# Exploring health-related quality of life in eating disorders by a cross-sectional study and a comprehensive review

**DOI:** 10.1186/1471-244X-14-165

**Published:** 2014-06-04

**Authors:** Monica Baiano, Pierandrea Salvo, Pierluigi Righetti, Lucia Cereser, Erika Baldissera, Ilenia Camponogara, Matteo Balestrieri

**Affiliations:** 1 Centre for Weight and Eating Disorders, Azienda Socio-Sanitaria Locale n. 10 “Veneto Orientale”, Venice, Portogruaro, Italy; 2 Unit of Psychiatry, Dept. of Experimental and Clinical Medical Sciences, University of Udine, Udine, Italy; 3 Clinica Psichiatrica, Azienda Ospedaliero-Universitaria, P.le S.M. Misericordia 15, 34070 Udine, Italy

**Keywords:** Eating disorders, Anorexia nervosa, Bulimia nervosa, Binge eating, HRQoL, Quality of life, WHOQoL-BREF, SF-36, Review

## Abstract

**Background:**

People with eating disorders (ED) often report poor health-related quality of life (HRQoL), which is explicitly correlated to illness’ severity and its effects on cognitive performance. We aimed to analyze health-related quality of life (HRQoL) in subgroups of eating disorder (ED) patients by using the brief version of WHOQoL questionnaire (WHOQoL-BREF) before treatment administration. Moreover, in order to compare our findings with other published data, we carried out a comprehensive review of the literature on HRQoL in ED patients.

**Methods:**

Our review was carried out by means of an accurate data mining of PsychInfo and Medline databases and other available sources. In our cross-sectional study, eighty female ED patients (26 with bulimia nervosa, 33 with anorexia nervosa, 7 with binge eating disorder and 14 with ED not otherwise specified) completed the WHOQoL-BREF. HRQoL scores were compared among ED subgroups and clinical information (presence of previous contacts, length of illness, psychiatric comorbidity) was considered in the analysis.

**Results:**

Our review shows that with few exceptions ED patients have a poorer HRQoL than the healthy population of control and sometimes the mental component of HRQoL is the most involved dimension. Moreover, there are no differences in the HRQoL among ED groups, even if AN patients in some studies have a lower HRQoL scores. Furthermore, BED patients have a poorer HRQoL than obese patients who do not have binge episodes. Finally, all treatments were positively correlated with an improvement on general and specific QoL dimensions. In our sample, ED subgroups differed only for Psychological Health HRQoL scores (F = 4.072, df = 3; p = 0.01). No differences were found between inpatients and outpatients, treatment naïve and previously treated patients and patients with or without psychiatric comorbidity. Moreover, HRQoL scores were not correlated to length of illness within each ED subgroup.

**Conclusions:**

The analysis of the literature adds some relevant information on HRQoL in ED and may address the future research toward the exploration of specific questions. One of these may be the prominent role of Psychological Health domain in HRQoL, since our study confirms that this component is able to differentiate eating disorders.

## Background

According to the World Health Organization (WHO), QoL refers to “the individuals’ perceptions of their position in life in the context of the culture and value systems in which they live and in relation to their goals, expectations, standards and concerns” [1]. On the other hand, the concept of health-related QoL (HRQoL) encompasses those aspects of overall quality of life that can be clearly shown to affect health, either physical or mental. On the individual level, this includes physical and mental health perceptions and their correlates, while on the community level HRQoL includes resources, conditions, policies, and practices that influence a population’s health perceptions and functional status [2]. As reviewed by Engel and colleagues [3], most of studies investigating the HRQoL in eating disorder (ED) patients measured the effects of abnormal eating behavior on health status but not on HRQoL.

Specifically, the HRQoL is associated with the degree of illness-related disability and effects of medical interventions on perceived HRQoL [4,5]. Indeed, people with ED often report poor HRQoL [6], which is explicitly correlated to illness’ severity and its effects on cognitive performance [7,8]. Cognitive impairments may significantly reduce adherence to individualized rehabilitation programs, thus favoring chronicity and psychosocial deterioration [9]. Moreover, ED are frequently associated with comorbid psychiatric disorders, especially anxiety, somatoform and depressive disorders, which further prevent recovery and increase the probability of resistance to treatment efforts [10,11].

Hence, the assessment of HRQoL in ED is crucial to predict the clinical outcome in patients undergoing specific treatments, as well as the risk of relapse and recurrence [12]. Although some ED-specific instruments have been used to assess HRQoL [13], none of them offers a global viewpoint of patient’s perception of HRQoL. Conversely, the World Health Organization Quality of Life (WHOQoL) questionnaire [1] measures patient’s subjective awareness of illness related to physical and psychosocial dysfunctioning.

We wanted to investigate HRQoL in eighty female ED patients attending a Centre for Eating and Weight Disorders in Italy. In order to place our results within the frame of the current knowledge, we analyzed the literature and realized that a comprehensive review on the factors that can modulate HRQoL in eating disorders was lacking. So, we decided to implement a review on this topic, by selecting published papers written in English. The aims of the review were to:

•test the hypothesis that HRQoL is poorer in ED subjects than in healthy individuals

•explore the existence of different levels of HRQoL among different ED

•explore whether ED subjects have different impairments on mental vs. physical dimension of HRQoL

•explore the existence of differences in HRQoL between obese subjects and subjects with BED

•analyze the efficiency of ED-specific HRQoL instruments as compared with more general instruments

•analyze the efficacy on HRQoL of the treatment proposed for ED, including bariatric surgery.

Once that this review was completed, we were able to compare the results obtained in our sample with those emerging from all other studies. We were specifically interested in analyzing the HRQoL among patients with different eating disorders at the beginning of a psycho-nutritional rehabilitation program and to explore whether comorbid DSM-IV diagnoses, setting of care and history of previous treatment were involved in their perception of HRQoL.

## Methods

### Analysis of the literature

We firstly consulted other reviews on this topic, which revealed a number of shortcomings. The review by Engel et al. [3] is a narrative review focused only on the instruments used to measure the HRQoL in ED patients. The Authors identified four ED-specific HRQoL instruments (the 25-item EDQOL, the 40-item EDQLS, the 55-item HeRQUoLED and the 20-item QOL-ED) and discussed their relevance in the field of the treatment of ED.

A second review was carried out by Passarelli-Tirico et al. [14], who classified the papers according to the quality of the design, as defined by the Australian governative Agency NHMRC. The shortcomings of this review are that it is not in English and does not include the results of HRQoL analyses.

The third review was carried out by Jenkins et al. using a narrative approach [15]. This is a comprehensive review, which allows to enter into the details of the papers and to analyze specific issues such as the HRQoL associated with subclinical ED pathology, BMI, or purging and other compensatory behaviors. However, it does not include a general table to compare the studies and does not attempt to systematically describe the research design, the setting or the type of assessment used.

In our comprehensive review we aimed to include papers that used validated HRQoL instrument and to classify patients according to definite diagnoses. Since this was not always possible, we considered also papers that assessed the patients according to eating dimensions or to subclinical levels of disordered eating.

Accurate data mining was conducted by consulting PsychInfo and Medline databases and other available sources, such as the references included in the papers reviewed. The databases were examined thoroughly using the keywords “quality of life, QoL, HRQoL, functional impairment, eating disorders, anorexia nervosa, bulimia nervosa, binge eating disorder”.

The accuracy of the assessment among the papers included was quite variable, ranging from simple ED questionnaires to clinical or semi-structured interviews. Most frequently, the assessment was conducted with instruments of the Eating Disorders Examination (EDE) series, either questionnaire or interview. Other structured instruments were the SCID or the PRIME-MD. In reporting the data means and SD where not shown, since these statistics were not always inferable (for example, they were shown in figures and not in tables) or else were too many (one statistic for each ED and for each subscale) to be included in the table. Moreover, apart from studies using SF-36, most statistics were taken with miscellaneous instruments and thus were not comparable.

Finally we excluded those papers that analyzed the HRQoL in obese patients without attempting to assess the presence of a whatsoever ED within the sample.

### Procedures of the cross-sectional study

We recruited eighty female ED patients (mean age: 28.24 ± 11.28 SD; range: 13–61 years) attending the Centre for Eating and Weight Disorders of the local Socio-Health Unit (ASSL “Veneto Orientale”, Portogruaro, Italy). This Center is a highly specialized unit, where treatment-resistant ED subjects are referred from a vast area including health districts both of Veneto and of Friuli regions. On the other hand also subjects living in the nearby surroundings can be accepted. Patients’ diagnoses were established using the DSM-IV-TR criteria for Anorexia Nervosa, Bulimia Nervosa, and Binge Eating Disorder. Subjects who did not meet full diagnostic criteria were classified as Eating Disorder Not Otherwise Specified. Comorbidities were assessed taking in consideration the medical history, and confirming their current presence with the clinical assessment. Exclusion criteria included the presence of clinically relevant multiorganic disorder or cerebral organic impairment and patients not completing the assessment for language barriers. All patients were informed about the main objectives of our search and all signed written informed consent.

Our study was carried out in compliance with the Helsinki Declaration, using the database of Centre for Weight and Eating Disorders of Portogruaro, a credited agency of the Regional Health System. Patients gave their written consent to the use of their data for both clinical and research purposes at the time of their first contact with the Centre. Since no new instrument or investigation were used, a specific request to the local ethics committee was not advanced, being valid the principle of the availability for research of the data gathered in the clinical practice.

All participants were assessed for self-perception of HRQoL using the WHOQoL-BREF [1] at the beginning of treatment. This questionnaire is a 26-item short version of the WHOQoL scale measuring the subjective perception of quality of life associated with physical and psychological health, social performance and environment’s characteristics. The Physical Health scale specifically investigates activity of daily living, dependence on drugs and medical supports, level of energy, mobility, pain and discomfort, sleep and rest, work capacity. The Psychological Health scale explores body image and appearance, positive and negative feelings, self-esteem, spirituality and personal believes, cognitive functioning. The Social Relationship scale measures quality of social relationships, social support and sexual activity. Finally, the Environment scale evaluates individual’s socio-economic conditions (as financial resources, freedom, physical safety and security) and the availability of facilities in living and working context (accessibility to health and social care, home environment, chances for acquiring new information and skills, recreation and leisure activities, physical environment and transport).

Items are scored on a Likert five-point scale, were “1” means severe discontent and “5” great approval; patients had to evaluate their quality of life considering the two weeks preceding the administration of the questionnaire. The mean score of items within each scale was multiplied by 4 in order to be comparable to those of the 100-items WHOQOL scale.

Finally, height and weight were measured to calculate the body mass index (BMI: kg/m^2^).

### Statistical analysis

Data were analyzed using SPSS Version 16.0 for Windows [16] and STATA 10.0 [17]. The statistical significance (alpha) was set at p < 0.05. Firstly, data were inspected for normality using the Shapiro-Wilk test.

Socio-demographic and clinical variables were compared among ED subgroups using the Kruskal-Wallis or Fisher’s exact test, as appropriate. Finally, an analysis of covariance (multivariate ANCOVA) was used to examine whether 1) there were significant differences between ED subgroups, after controlling for age and length of illness; 2) there were differences for HRQoL scores between ED inpatients and outpatients, ED patients with and without history of previous treatment, patients with and without comorbid DSM-IV diagnosis, controlling for ED diagnosis. Bonferroni’s correction for multiple comparisons was also applied.

Finally, Pearson’s correlation analysis was performed to explore the relationship between HRQoL scores and length of illness.

## Results

As it shown in Table 1, three main typology of study could be identified in the literature. The first one is the population survey, carried out with different methods and with dissimilar response rates. Some were postal surveys, others were conducted via telephone, and further more used a face to face interview. A second type of design is the cross-sectional analysis of samples of subjects attending outpatients ED centers or of patients waiting for a gastric by-pass surgery. The third type of design is the cohort prospective study. Within this category, there is one single multi-wave survey and a number of papers focused on treatments: within the latter studies, we can enumerate RCT studies with drugs, one study with a specific psychotherapy (CBT) treatment, studies on patients who attended a nutrition program or other unspecified programs in ED centers and one study which evaluated a gastric by-pass surgery intervention.

**Table 1:** Review of the literature on QoL in eating disorders subjects

**Study**	**Design, treatment**	**Setting, characteristics of whole sample**	**ED pts studied**	**Control or reference group(s)**	**Assessment**	**HRQoL Instrument**	**Results on general and specific HRQoL measures (> means superior QoL)**
**Surveys**							
[12] Spitzer et al., 1995	Two-stage survey	1000 primary care pts	30 BED (with 84% psychiatric comorbidity)	614 without Mental Disorders (MD)	PRIME-MD	SF-20	• BED (as well other MD) < No-MD.
							• BED < non-BED on social functioning and bodily pain
[18] Hay 2003	Comunity survey	3010 out of 4400 (response rate 70%)	78 BED, 60 subj with extreme weight control behaviors (EWCB)	Australian normative sample	EDE	SF-36, AQoL	• BED and EWCB < Normative on MCS
[19] Doll et al., 2005	Postal survey	1439 out of 3750 students (response rate 42%)	83 (5.8% of respondents) with ED (54 BN, 22 BED, 7 AN)	1148 non-ED subjects	Ad-hoc questionnaire based on DSM-IV	SF-36	• ED < non-ED subj on MCS, but not on PCS.
							• BN and BED < non-ED subj on MCS
[20] Herpetz-Dahlmann et al., 2008	Community survey	1895 adolescents	400 ED not classified according DSM criteria	1495 non-ED	SCOFF, not confirmed by interview	KINDL-R	• ED < non-ED
[21] Vallance et al., 2011	Cross-sectional study	Recruitment at the university campus and in newspapers	103 women with 2+ episodes of binge eating per month	109 women with <2 episodes of binge eating per month	EDE-Q, EDI-2	SF-36	• High frequency of binge eating predicted poorer QoL
[22] Mond et al., 2012	Two stage community study	324 interviewed at the second stage	159 ED (30 BN, 20 BED, 109 EDNOS)	232 healthy women from different survey in same area	EDE-Q + EDE	SF- 12, WHOQOL-BREF	• ED < Healthy women on MCS
[23] Mitchison et al., 2013	Population survey	3034 out of 5000 selected (response rate 60.7%)	89 AN (2.9% of respondents)	2945 subj with no history of AN	Interview based on EDE	SF-36	• AN < other subj on most domains, including MCS.
							• Subj with history of AN < other subj on MCS but not on PCS.
							• Impairment on social functioning and role limitations greater with current ED symptoms
**Cross-sectional studies**							
**ED patients**							
[24] Keilen et al., 1994	Cross-sectional study	ED outpatient center	126 ED (52 AN, 74 BN)	98 males with angina; 122 hearth transplant candidates; 54 cystic fibrosis pts; 91 students	Clinical interview (DSM-III-R)	NHP	• Specific differences between ED and pts with organic diseases
[10] Padierna et al., 2000	Cross-sectional study	ED outpatient center	197 ED (116 AN, 64 BN, 17 BED)	Norm-based scoring of Spanish general population	Clinical interview (DSM-IV)	SF-36	• ED pts < normative population.
							• BED < other ED on physical functioning
[25] Gonzalez-Pinto et al., 2004	Cross-sectional study	ED outpatient center	47 AN	No control	SCID I and II	SF-36	• Predictive variables for PCS: poor outcome in previous year, comorbidity and female gender.
							• For MCS: comorbidity and purging behaviors
[7] De La Rie et al., 2005	Cross-sectional study	Mixed: population via advertisements and ED centers	156 ED pts (44 AN, 43 BN, 69 EDNOS) and 148 former ED pts	Dutch normative population and 591 Mood Disorders (MD) pts	DSM-IV diagnosis based on EDE-Q + BMI and menstrual status	SF-36	• No diff among ED groups.
							• ED < normative.
							• Former ED < normative. ED < MD
[11] Mond et al., 2005	Cross-sectional study	Pts referred to ED treatment program	87 ED pts (34 AN, 40 BN, 10 EDNOS)	495 general population women	Clinical assessment + EDE-Q	WHOQoL-BREF	• ED pts < normative subjects.
							• Restricting AN pts > other patient groups.
							• BED < other patients on PCS
[26] Engel et al., 2006	Cross-sectional study to validate EDQOL	538 recruited sample of student	155 ED, 56 diet/exercise	Validation across groups, including 327 non-ED subj	SCID + EDE + EAT-26	EDQOL, SF-36	• All EDQOL subscale scores differed between groups, with greater impairment in ED pts.
							• EDQOL more sensitive than SF-36 when predicting group status (ED vs. diet/exercise)
[27] De La Rie et al., 2007	Cross-sectional study	Mixed: population via advertisements and ED centers	146 ED pts (44 AN, 43 BN, 59 EDNOS)	146 former ED	DSM-IV diagnosis based on EDE-Q + BMI and menstrual status	SEIQOL	• ED with poor QoL on all life domains.
							• Former ED pts > ED pts on most domains (but ratings just above average)
[28] Latner et al., 2008	Cross-sectional study	ED outpatient center	11 AN, 5 BN, 3 BED, 30 EDNOS, 4 non-ED	New Zealand normative population	EDE-Q	SF-36	ED < normative on MCS.
							• QoL general and PCS predicted by subjective bulimic episodes
[29] Bamford & Sly, 2010	Cross-sectional study	ED outpatient center	156 ED (80 AN, 40 BN, 36 EDNOS)	Comparison across ED groups	EDE-Q	EDQOL	• AN < BN and EDNOS on psychological and physical/cognitive domains
Baiano et al., present study	Cross-sectional study	ED center (in- and out-patients)	80 ED (26 BN; 33 AN; 7 BED; 14 EDNOS)	Comparison across ED groups	Clinical interview (DSM-IV)	WHOQoL-BREF	• No diff among ED groups. EDNOS > other groups on psychological health QoL
**Obese patients**							
[30] Hsu et al., 2002	Cross-sectional study	37 subj awaiting GBP	9 BED	28 non-BED	EDE, TFEQ, SCID-IV	SF-36	• BED < non-BED
[31] De Zwaan et al., 2002	Cross-sectional study on pre and post-operative patients	78 obese surgical pts	78 obese (9 BED) after GBP surgery	110 preoperative control group (19 BED)	Phone interview + MFED + QWEPR	SF-36	• Postoperative pts > preoperative pts.
							• Postoperative pts < US norm values on PCS
[32] Masheb & Grilo, 2004	Cross-sectional study	Pts undergoing a medical school based ED treatment	94 BED	US normative population and Obeses without binges (n = 312)	Clinical interview (DSM-IV)	SF-36	• BED < normative.
							• BED < non-BED on PCS
[33] Kolotkin et al., 2004	Cross-sectional study	530 obese candidates to residential modification program	95 BED	435 non-BED	Questionnaire on Eating/Weight Patterns; BDI; SC90-R	IWQOL-Lite	• BED = non-BED when other variables are considered
[34] Rieger et al., 2005	Cross-sectional study within a RCT	118 treatment-seeking obese subj	56 BED	62 non-BED	EDE, PRIME-MD	IWQOL-Lite	• BED < non-BED on total scale, but not on physical function subscale
[35] Colles et al., 2008	Cross-sectional study	180 bariatric surgery candidates, 93 participants to a weight loss support group, 158 community respondents	38 BED, 46 subj with feelings of loss of control (LOC) during binge episodes	307 non-binge eaters	QEWP-R + semistructured interview or phone interview	SF-36	• BED < non-BED on MCS
[36] Folope et al., 2012	Cross-sectional study	130 obese in clinical nutrition center	73 ED	57 non-ED	SCOFF-F + BULIT, not confirmed by a diagnosis	QOLOD	• ED < non-ED, globally and on psychological dimension
[37] Ranzenhofer et al., 2012	Cross-sectional study	158 obese adolescents selected for weight-loss treatment	35 binge eating (6 proper BED)	123 non-binge eaters	EDE	IWQOL-A	• Binge eating < no-binge eating.
							• Girls with binge eating < boys with binge eating
**Cohort studies**							
**Survey**							
[38] Wade et al., 2012	Longitudinal multi-wave survey	9,688 population of women	2223 ED	7465 non-ED	Ad-hoc questionnaire, EDE-Q, not confirmed by interview	SF-36	• ED < non-ED, globally and on PCS and MCS
**ED outpatient treatments**							
[39] Padierna et al., 2002	2 years cohort study	ED outpatient center	131 ED (90 AN, 41 BN)	Spanish normative population	Clinical interview (DSM-IV)	SF-36	• Improvement in PCS and social function, followed by MCS.
							• Scores after 2 years still below normative population.
							• Severity of ED affected improvement
[40] Muñoz et al., 2009	Cohort study (baseline, after 1 year)	358 subj in treatment programs in Health centers	61 AN, 47 BN, 245 EDNOS	305 general population women	Clinical interview (DSM-IV)	HeRQoLED, SF-36	• ED < general population.
							• After 1 year PCS improved but not MCS.
							• AN < other ED at baseline, and smaller improvements after 1 year
[41] Adair et al., 2010	Cohort study (baseline, 3 and 6 months follow-ups) to validate EDQLS	ED treatment programs	130 ED pts (56 AN, 39 BN, 35 EDNOS)	QoL measures at different point in time	Clinical	EDQLS, Quality of Life Inventory, SF-12	• EDQLS total scores increased at 3 and 6 months.
							• EDQLS responsiveness exceeded that of other QoL instruments
**Specific treatments**							
[42] Marchesini et al., 2002	Intervention study (3–5 months CBT)	96 obese enrolled in a CBT program	46 BED (44% of sample)	76 untreated controls in waiting list	Interview, BES, EDE	SF-36	• Treated subjects improved in QoL, with improvement larger in BED, both in general scores and in PCS and MCS
[43] Nickel et al., 2005	Intervention study (10 weeks RCT with topiramate)	60 BN women recruited through advertisements	30 BN on topiramate	30 BN on placebo	SCID-I and SCD-II	SF-36	• Topiramate improved QOL to a greater extent than placebo
[44] Wilfley et al., 2008	Intervention study (24 weeks RCT with sibutramine)	304 BED recruited through advertisements	152 BED on sibutramine	152 BED on Placebo	EDE	IWQOL-Lite	• Sibutramine efficaciuos on psychopathology but not on QoL
**Residential treatments**							
[45] Abraham et al., 2006	Cohort study (baseline, at discharge and 12 months follow-up)	In-patient in ED center	206 ED pts (71 AN, 55 BN, 80 EDNOS)	35 subj without diagnosis	Clinical interview	EEE-C QOLscores, SF12	• QoL improved during inpatient treatment and between admission and 12 months after discharge.
							• AN, BN and EDNOS < no diagnosis.
							• Specific differences among ED groups on some dimensions
[46] McHugh, 2007	Prospective residential cohort study	ED residential center	65 AN (33 high Readiness for Change - RFC - females vs. 32 low RFC females)	Comparison between high- and low-RFC	Clinical interview (DSM-IV)	SF-36 v2	• Participants’ QoL below US average.
							• 81% discharged below the US average.
							• No diff between RFC and non-RFC
**Surgical interventions**							
[47] Green et al., 2004	Cohort study	65 surgical (GBP) pts	33 BED	32 non-BED	ED-SCID, QWEP-R	SF-36	• QoL improved from pre-surgery to post-surgery.
							• BED < non-BED on social functioning at pre-surgery and after 6 months postsurgery

Glossary of QoL instruments.

*AQoL*, assessment of quality of life.

*EDE-Q*, eating disorder examination questionnaire.

*EDQLS*, eating disorders quality of life.

*EEE-C QOL*, eating and exercise examination QOL.

*HeRQoLED*, health related quality of life for the eating disorders.

*IWQOL*, impact of weight on quality of life-lite.

Kindl-R**:** Revised German-language questionnaire to assess Health-Related Quality of Life in children and adolescents.

*NHP*, Nottingham health profile questionnaire.

*QOLOD*, quality of life, obesity and dietetics rating scale.

*SEIQOL*, schedule for the evaluation of individual quality of life.

*SF-36*, short form (36) health survey.

*WHOQoL-Bref*: brief version of the world health organization quality of life questionnaire.

An important issue is the preference accorded from most studies to the SF-36 scale or its derivatives for the assessment of HRQoL. The SF-36 is a well-known but unspecific instrument for the study of ED and allows a distinction between mental component symptoms (MCS) and physical component symptoms (PCS). Other generic instruments include for example the WHO scale WHO-BREF, which was used in a few studies. More specific instruments are EDQoL, the HeRQUoLED, the EDQLS, the IWQOL, but only few studies adopted them.

The main evidence that comes out from our review is that with few exceptions ED patients have a poorer HRQoL than the healthy population of control. Frequently MCS is the most involved dimension, which means that ED patients are particularly vulnerable to impairment in the QoL because of their psychic difficulties, being the physical component less harming. But this is not always the rule.

Another piece of evidence is that frequently there are no differences in the HRQoL among ED groups. AN patients in some studies have a lower HRQoL and this is particularly evident when a specific instrument is used, like the EDQoL.

Among potential surgical patients waiting for a GBP, most - but not all - studies conclude that BED patients have a poorer HRQoL than obese patients who do not have binge episodes.

Finally, when we consider the efficacy of the treatment on HRQoL measures, all treatments were positively correlated with an improvement on the general and, when examined, specific QoL dimensions. However, when compared with healthy individuals, their HRQoL remained still below the norm.

### Characteristics of our sample

All relevant socio-demographic and clinical data of our cross-sectional study are reported on Tables 2 and 3. Fifty-nine out of 80 subjects were outpatients (73.8%) whereas the remaining 21 (26.3%) were inpatients. Among all patients, 33 (41.3%) were affected by AN (mean age: 26.1 years ±10.6 SD), 26 (32.5%) were affected by BN (mean age: 27.5 years ±8.0 SD), 7 were diagnosed as BED subjects (8.8%) (mean age: 41.6 years ± 14.0 SD) and 14 patients suffered from EDNOS (17.5%) (mean age: 28.1 years ± 13.3 SD). In addition, 14 patients (11 inpatients and 3 outpatients) fulfilled DSM-IV criteria for other ED: two for Borderline Personality Disorder (2.5%), nine for Major Depressive Disorder (11.3%) and one each for Bipolar Disorder, Alcohol Abuse and Social Phobia. Forty-six patients had a history of previous therapies for ED (25 inpatients and 21 outpatients) whereas the remaining 34 subjects were all treatment-naïve ED inpatients.

**Table 2:** Socio-demographic and clinical characteristics of ED patients: continuous variables

	**AN (N = 33)**	**BN (N = 26)**	**BED (N = 7)**	**EDNOS (N = 14)**	**Kruskal-Wallis test**	**Significance**
**Age (years)**	26.09 ± 10.59	27.46 ± 7.96	41.57 ± 14.01	28.07 ± 13.28	χ^2^ = 8.293	p = 0.040
**BMI (kg/m**^**2**^**)**	15.97 ± 1.88	21.99 ± 5.17	32.40 ± 8.26	24.73 ± 7.29	χ^2^ = 54.916	p < 0.001
**Age at onset (years)**	19.21 ± 6.93	17.92 ± 5.37	36.14 ± 15.40	22.57 ± 13.20	χ^2^ = 9.585	p = 0.022
**Age at first evaluation (years)**	26.06 ± 10.61	27.39 ± 8.00	41.57 ± 14.01	28.07 ± 13.28	χ^2^ = 8.198	p = 0.040
**Lenght of illness (years)**	6.88 ± 7.27	9.54 ± 7.20	5.43 ± 7.00	5.50 ± 9.97	χ^2^ = 9.456	p = 0.020

Mean ± SD.

**Table 3:** Socio-demographic and clinical characteristics of ED patients: categorical variables

	**AN (N = 33)**	**BN (N = 26)**	**BED (N = 7)**	**EDNOS (N = 14)**	**Fisher’s exact test**
**Occupation**					P = 0.070
Yes	7 (21.2%)	13 (50%)	4 (57.1%)	7 (50%)	
No	26 (78.8%)	13 (50%)	3 (42.9%)	7 (50%)	
**Marital status**					P < 0.001
Unmarried	29 (87.9%)	21 (80.7%)	1 (14.3%)	11 (78.6%)	
Married or coupled	3 (9.1%)	2 (7.7%)	5 (71.4%)	3 (21.4%)	
Divorced or separated	1 (3.0%)	3 (11.5%)	1 (14.3%)	0	
**Educational level**					P = 0.093
Primary school	16 (48.5%)	5 (19.2%)	5 (71.4%)	6 (42.9%)	
High school	11 (33.3%)	17 (65.4%)	2 (28.6%)	5 (35.7%)	
University	6 (18.2%)	4 (15.4%)	0	3 (21.4%)	
**Living conditions**					P = 1.000
Alone	3 (9.1%)	2 (7.7%)	0	1 (7.1%)	
With others	30 (90.9%)	24 (92.3)	7 (100%)	13 (92.9%)	
**Patients completing education**					P = 0.810
Yes	14 (42.4%)	8 (30.8%)	2 (28.6%)	5 (35.7%)	
No	19 (57.6%)	18 (69.2%)	5 (71.4%)	9 (64.3%)	
**Setting**					P = 0.005
Inpatients	14 (42.4%)	7 (26.9%)	0	0	
Outpatients	19 (57.6%)	19 (73.1%)	7 (100%)	14 (100%)	
**Previous treatment for ED**					P < 0.001
Yes	27 (81.8%)	14 (53.8%)	1 (14.3%)	4 (28.6%)	
No	6 (18.2%)	12 (46.1%)	6 (85.7%)	10 (71.4%)	
**DSM-IV comorbidity**					P = 0.431
Yes	7 (21.2%)	6 (23.1%)	0	0	
No	26 (78.8%)	20 (76.9%)	7 (100%)	14 (100%)	
**Illness’ awareness**					P = 0.548
Yes	21 (63.6%)	16 (61.5%)	5 (71.4%)	6 (42.9%)	
No	12 (36.4%)	10 (38.5%)	2 (28.6%)	8 (57.1%)	

ED subgroups significantly differed for age (χ^2^ = 8.293, p = 0.040), BMI (χ^2^ = 54.916; p < 0.001), age at onset (χ^2^=; p = 0.02), age at first evaluation (χ^2^ = 8.198; p = 0.04) and length of illness (χ^2^ = 9.585; p = 0.022). Also, differences among ED subgroups were found for marital status (p < 0.001), setting (p = 0.005), history of previous treatments (p < 0.001) but not for educational level or living conditions, occupational status, proportion of patients completing their studies and DSM-IV psychiatric disorder comorbidity (p > 0.05).

Significant differences emerged when considering the presence of a history of previous treatments for ED, both between ED inpatients (100% untreated) and outpatients (54.3% untreated) (p < 0.001) and between patients with psychiatric comorbidity (14.2% untreated) and without psychiatric comorbidity (48.5% untreated) (p = 0.03). In opposition, no difference was detected for psychiatric comorbidity between inpatients (81% no comorbidity) and outpatients (85.7% no comorbidity) (p > 0.05).

### HRQoL scores in our sample

All ED patients’ HRQoL scores were below normative population values (Table 4). Results of ANCOVA showed that ED subgroups differed only for Psychological Health QoL scores (F = 4.072, d.f. = 3; p = 0.01; ANCOVA, age and length of illness as covariates). No differences were found for all HRQoL scores when patients were subgrouped for BMI levels (underweight: < 18.5 BMI; normal weight: 18.5-24 BMI; overweight/obese: ≥ 25) (p > 0.05, ANOVA or Kruskal-Wallis test, as appropriate). As to respect to illness’ awareness, differences among ED subgroups did not reach statistical significance (p > 0.05, Fisher’s exact test).

**Table 4:** HRQoL scores among ED subgroups

**HRQoL domains**	**ED diagnosis**	**Mean SD**	**Statistics**	**Significance**
**Physical**	*AN*	12.69 ± 3.09	F = 0.638	p = 0.593
	*BN*	12.39 ± 3.40		
	*BED*	11.75 ± 2.21		
	*EDNOS*	13.59 ± 3.55		
**Psychological**	*AN*	11.28 ± 1.28	F = 4.390	p = 0.007^
	*BN*	11.04 ± 1.45		
	*BED*	10.52 ± 0.94		
	*EDNOS*	12.56 ± 1.96		
**Social**	*AN*	10.87 ± 3.86	F = 0.772	p = 0.513
	*BN*	10.76 ± 3.01		
	*BED*	11.43 ± 4.21		
	*EDNOS*	12.47 ± 4.10		
**Environment**	*AN*	12.35 ± 2.55	F = 0.383	p = 0.766
	*BN*	12.80 ± 2.09		
	*BED*	13.14 ± 3.36		
	*EDNOS*	12.96 ± 2.02		

No differences for HRQoL scores were found between inpatients and outpatients, as well as treatment naïve or previously treated patients (p > 0.05). Also, ED patients with a DSM-IV comorbid diagnosis did not differ from those without psychiatric comorbidity for HRQoL scores (p > 0.05; ANCOVA, ED diagnosis as covariate). Ultimately, HRQoL scores were not significantly correlated to length of illness within each ED subgroup (p > 0.05, Pearson’s correlation analysis).

## Discussion

The main evidences coming out from our review are the followings:

•the surveys confirmed that ED patients have a poorer HRQoL than the general population and this is evident in particular when considering the mental component of the quality of life;

•among the obese patients, quite invariably the BED had a poorer HRQoL than non-BED subjects. This is an important piece of information, since it supports the idea that there is a need of specific treatments for BED, which are not simply an adaptation of general nutrition intervention;

•in the cohort studies, we can distinguish general programs aimed to treat the ED from specific psychotherapies and RCT with psychotropic drugs. Even if the published data seem to reassure that many treatments are able to improve the HRQoL, we remain with the doubt whether the amelioration was due to some specific characteristics of the treatment or was a generic result of the caring of these patients. Much has to be done in order to respond to the question “what aspect of treatment improve HRQoL of ED patients?”.

Furthermore, we must highlight the fact that most studies used general scales for measuring the HRQoL, and in particular the SF-36. Since much fewer information comes from studies that used specific scales (such as EDQOL), so far it is not possible to draw conclusion on the advantages of the latter scales. On the other hand, the analysis of Table 1 may suggest that the use of ED specific scales is able to increase the probability to discover subtle differences in HRQoL among different ED groups or between pre- and post-treatment scores.

When we consider our cross-sectional study, we must admit that our sample is of relatively small size, even if it is on average with other studies. To overcome this limit we used a non-parametric test (Kruskall-Wallis test), which in principle does not put limits in the sample size.

The main difference of our research as compared with other studies is that we included both inpatients and outpatients. It is worth noting that over 40% of outpatients, but none of the inpatients, had a history of previous treatments. If we assume that inpatients have a more severe illness, we may speculate that these patients did not seek help because of their low illness’ awareness and, in addition, that primary care physicians did not identify an ED in these subjects, although they were presenting important weight alterations, repetitive vomiting or bingeing [48]. Therefore, specific interventions were dramatically delayed until the need for an admission was necessary.

Additionally, over 85% of patients with comorbid psychiatric diagnoses reported a history of previous treatment for ED, in comparison to 50% of patients without coexisting psychiatric diagnoses. These results may indicate that the presence of comorbidity increased the probability to be treated earlier. We know from previous surveys that ED are frequently comorbid with Major Depressive Disorder, Anxiety Disorders and Substance or Alcohol Abuse [49] and also that ED relapses and successive hospitalizations reduce the response to treatment of comorbidity [50,51]. We may conclude that the comorbidity of severe ED psychopathology with other psychiatric disorders contributed to chronicity of illness and should be considered as a prognostic factor influencing long-term outcomes of rehabilitation programs and increasing financial dependence on society [52-54].

As far as HRQoL is concerned, our study confirmed results obtained by previous investigations, demonstrating that ED patients experienced reduced HRQoL. Surprisingly, ED inpatients and outpatients did not differ as for HRQoL scores. Most ED inpatients in our sample were AN, thus confirming that anorexic behaviors implicate a high risk of organic complications and require a large amount of intensive medical care [55]. It is possible that AN inpatients did not report greater limitations of QoL in comparison to the other subgroups, because of a poor perception of psychosomatic distress [56,57]. Moreover, we found no differences in HRQoL between ED patients with and without a comorbid DSM-IV diagnosis, as well as between patients with and without a history of previous treatments. Furthermore, HRQoL scores were not significantly correlated to length of illness within each ED subgroup. Taken together, all these negative results may indicate that our patients were impaired in their quality of life independently from their history and current severity of illness.

## Conclusions

Finally, when we consider the specific domains of HRQoL involved, the only difference among the ED groups was present in the Psychological component, where patients with EDNOS reported the highest HRQoL scores and patients with BED the lowest ones. In fact EDNOS patients represent a heterogeneous category of subjects with eating behaviors generating a relatively modest distress [58,59], and our EDNOS patients seem to confirm this rule. Also the data on our patients with BED are in agreement with previous studies, which documented great health dissatisfaction, increased risk of main medical disorders [60], difficulties in regulating emotions [61] and concurrence of personality traits, as well as comorbidity with mood and anxiety disorders [62,63] in binge eaters. All together, these last results may indicate the need in the future studies to focus the attention in particular on the characteristics of the psychological domain of HRQoL in eating disorders.

## Competing interests

In the past five years none of the Authors received reimbursements, fees, funding, or salary from an organization that may in any way gain or lose financially from the publication of this manuscript, either now or in the future. None of the Authors hold any stocks or shares in an organization that may in any way gain or lose financially from the publication of this manuscript, either now or in the future. There are not non-financial competing interests (political, personal, religious, ideological, academic, intellectual, commercial or any other) to declare in relation to this manuscript.

## Authors’ contributions

MoB and PS are the principal investigators of the cross-sectional study and MoB performed the statistical analysis of the study. PR, LC, EB and IC participated in the design of the cross-sectional study and collected the data. They were involved in the critical revision of the manuscript. MaB participated in the design of the cross-sectional study and carried out the review of the literature. He also drafted the manuscript, and takes the main responsibility for the interpretation of data. All authors read and approved the final manuscript.

## Pre-publication history

The pre-publication history for this paper can be accessed here:

http://www.biomedcentral.com/1471-244X/14/165/prepub
